# Elevated Carbon Dioxide and Nitrogen Impact Wheat and Its Aphid Pest

**DOI:** 10.3389/fpls.2020.605337

**Published:** 2020-12-01

**Authors:** Eva Carreras Navarro, Shu Kee Lam, Piotr Trębicki

**Affiliations:** ^1^Agriculture Victoria, Horsham, VIC, Australia; ^2^School of Agriculture and Food, Faculty of Veterinary and Agricultural Sciences, The University of Melbourne, Parkville, VIC, Australia

**Keywords:** climate change, food security, carbon dioxide, fertilizer, agriculture, wheat pest

## Abstract

The rise in atmospheric carbon dioxide (CO_2_) generally increases wheat biomass and grain yield but decreases its nutritional value. This, in turn, can alter the metabolic rates, development, and performance of insect pests feeding on the crop. However, it is unclear how elevated CO_2_ (eCO_2_) and nitrogen (N) input affect insect pest biology through changes in wheat growth and tissue N content. We investigated the effect of three different N application rates (low, medium, and high) and two CO_2_ levels (ambient and elevated) on wheat growth and quality and the development and performance of the bird cherry-oat aphid, a major cereal pest worldwide, under controlled environmental conditions. We found that eCO_2_ significantly decreased total aphid fecundity and wheat N content by 22 and 39%, respectively, when compared to ambient CO_2_ (aCO_2_). Greater N application significantly increased total aphid fecundity and plant N content but did not offset the effects of eCO_2_. Our findings provide important information on aphid threats under future CO_2_ conditions, as the heavy infestation of the bird cherry-oat aphid is detrimental to wheat grain yield and quality.

## Introduction

The current atmospheric carbon dioxide (CO_2_) concentration of 414 ppm (NOAA, 2020) is projected to double by the end of this century (RCP 8.5; Stocker et al., 2013), resulting from fossil fuel combustion and deforestation (IPCC, 2014). Climate models predict an increased occurrence of extreme temperature, rainfall, and drought events under future climatic conditions (IPCC, 2012), therefore, threatening the resilience of current food production systems (Campbell et al., 2016). Projections indicate that feeding a world population of 9.7 billion by 2050 (United Nations, 2019) will require doubling the current food production (Mbow and Rosenzweig, 2019). Thus, meeting the future food demand is considered a major challenge in the twenty-first century (Mbow and Rosenzweig, 2019).

Wheat (*Triticum aestivum* L.) is one of the most important cereal crops produced and consumed worldwide. It is a main source of carbohydrate in North America, Australia, Europe, the Middle East, Asia, and North and Sub-Saharan Africa (Awika et al., 2011; Shewry and Hey, 2015), and provides approximately 20% of the protein in human diet (Wang et al., 2013). Wheat has shown to be highly responsive (11.8–38% biomass increase) to CO_2_ fertilization (Jablonski et al., 2002; Högy et al., 2009; O’Leary et al., 2015; Fitzgerald et al., 2016). However, elevated CO_2_ (eCO_2_) also decreases the protein content and nutritional quality of wheat-derived products through decrease in plant nitrogen (N) content (Taub et al., 2008; Myers et al., 2014). A potential adaptation strategy to maintain the nutritional quality of wheat is optimizing the use of nitrogen fertilizer.

There are a number of biotic stresses behind crop losses worldwide, among which insect pests are of high importance. Despite the extensive use of insecticides, the total loss of food crops attributed to insect pests is estimated at 30–40% (García-Lara and Saldivar, 2016). In grain crops, such as rice, maize, and wheat, insect pests are currently responsible for 5–20% yield loss (Deutsch et al., 2018). Aphids are among the most important cereal pests worldwide, inflicting economic damage directly through feeding and through the spread of viruses. In temperate regions, the bird cherry-oat aphid, *Rhopalosiphum padi*, is one of the most important cereal pests (Morales-Hojas et al., 2020; Trębicki, 2020). It is distributed in all wheat growing regions worldwide and is the main vector of *Barley yellow dwarf virus* (BYDV), responsible for significant losses in cereal yield and quality (Smith and Sward, 1982; Jones and Naidu, 2019).

Climate change will impact aphid population size, migration activity, and distribution (Luck et al., 2011; Ryalls and Harrington, 2016; Trębicki et al., 2017; Deutsch et al., 2018; Trębicki and Finlay, 2019; Trębicki, 2020). In particular, eCO_2_ has shown to increase aphid metabolic rates and, thus, feeding behavior (Robinson et al., 2012; Trębicki et al., 2016), and to alter aphid development and fecundity through changes in host biochemistry (Johnson and Jones, 2017; Johnson and Züst, 2018; Trębicki and Finlay, 2019; Moreno-Delafuente et al., 2020). There are contrasting findings in terms of the effect of eCO_2_ on aphid fecundity (Xing et al., 2003; Robinson et al., 2012; Oehme et al., 2013; Ryan et al., 2014; Ryalls and Harrington, 2016; Trębicki et al., 2016; Moreno-Delafuente et al., 2020), as it can be highly species/host specific (Hughes and Bazzaz, 2001). In terms of aphid development time, the findings can vary; for example, eCO_2_ did not affect the development time of *R. padi* reared on wheat (Trębicki et al., 2016) but significantly increased that of *Myzus persicae* reared on bell pepper (Dáder et al., 2016).

Despite its positive impact on crop production, increased N inputs have shown to increase insect populations by improving the nutritional quality of host plants (Cisneros and Godfrey, 2001; Aqueel and Leather, 2011), thereby increasing the damage of insect pests. In a meta-analysis, higher N inputs improved the performance of herbivore insects reared on broadleaf plants and conifers (Li et al., 2016). Greater N fertilizer application rates significantly increased the fecundity and longevity of the aphid *Hysteroneura setariae* when reared on rice (Jahn et al., 2005), and the fecundity and intrinsic rate of increase (maximum growth rate per individual for a population) of the cereal aphid *Metopolophium dirhodum* when reared on wheat (Gash, 2012). Moreover, the addition of N fertilizer increased the fecundity and decreased the development time of the bird cherry-oat aphid and the English grain aphid (Khan and Port, 2008).

Research has shown that changing climate conditions, mainly driven by the increase in CO_2_, will continue to alter the productivity and suitability of farmland (Mbow and Rosenzweig, 2019). As previously mentioned, increasing CO_2_ has a positive effect on C3 crop growth through carbon fertilization (Mitchell et al., 1993; Jablonski et al., 2002; Bloom et al., 2010; Taub, 2010; Lam et al., 2012; Ryan et al., 2014; O’Leary et al., 2015; Fitzgerald et al., 2016) but decreases the N content and nutritional quality (protein and macronutrients) of food crops (Taub et al., 2008; Myers et al., 2014; Vassiliadis et al., 2016). To replenish the extra N removed from grain cropping systems under eCO_2_ (Lam et al., 2012), increasing or optimizing N fertilizer application may be considered (Walker et al., 2017). The increase in N input, however, uncovers other challenges through its indirect impact on insect pests and diseases. Insect pests are known to display a strong response to plant N content (Cisneros and Godfrey, 2001; Khan and Port, 2008; Aqueel and Leather, 2011; Gash, 2012; Li et al., 2016). However, increased N fertilization and its indirect effect on insect-plant herbivore interactions under eCO_2_ are largely unknown (Sudderth et al., 2005; Ryan et al., 2014). Hence, this research investigates the interaction between current ambient and projected elevated CO_2_ levels [ambient CO_2_ (aCO_2_) = 400 ppm and eCO_2_ = 800 ppm] and three levels of N fertilization on wheat growth and quality, and on the development and performance of the bird cherry-oat aphid. We hypothesize that the effect of eCO_2_ on wheat growth and quality and consequently on aphid development and performance will be mitigated by greater N application rates.

## Materials and Methods

### Source of Bird Cherry-Oat Aphid

A single adult female bird cherry-oat aphid obtained from a field located near the Grains Innovation Park facility in Horsham, VIC, Australia was placed on an individual potted wheat (cv. Mace) plant. After 24 h, the female aphid and all its progeny except for a single nymph were removed. This nymph was placed on a new wheat plant in order to start a colony that was then used for the experiment. This clonal lineage was reared on wheat for over five generations prior to the experiment.

### Plant CO_2_ and Nitrogen Growing Conditions

All plants were grown in 0.5 L pots filled with 300 g of potting mix. Trace elements (Manutec PTY LTD) and different amounts of ammonium sulfate (Richgro Garden Products), corresponding to the three N treatments, were added into the nutrient-free potting mix, and then thoroughly mixed in a cement-mixer. The amount of ammonium sulfate applied to each N treatment was calculated based on wheat rooting depth, potting mix bulk density, and concentration of N in ammonium sulfate (21%; Speight, 2017). The low, medium, and high N treatments consisted of 141, 282, and 423 mg/100 g of ammonium sulfate, respectively.

Plants were grown in CO_2_-controlled plant growth chambers (Thermoline Scientific, TPG-1260) at a constant temperature of 20°C, and 16:8 D:L photoperiod (light intensity: 1000 μmol m^−2^ s^−1^ at plant canopy level, powered by five high pressure sodium 400 W lights and five 77 W incandescent lights). Plant growth chambers were set at either aCO_2_ (400 ppm) or eCO_2_ (800 ppm). Of the 48 potted plants that were sown for each N treatment, 24 were placed in a tray and grown at one of the two CO_2_ conditions. The same amount of water was applied to each tray daily. CO_2_ concentration and wheat plants were alternated between the chambers twice a week to eliminate any chamber-induced effect.

### Plant CO_2_ and Nitrogen Response Assessment

Plant growth parameters including plant height, tiller number, and chlorophyll content were measured on a weekly basis for 4 consecutive weeks. The change in plant height at different growth stages was used to evaluate plant growth (Demir et al., 2018), and the tiller number was used as an indirect measurement of biomass (Boe and Beck, 2008). Chlorophyll content was measured using SPAD chlorophyll meter (Soil Plant Analytical Development-502Plus, Konica Minolta, Japan), generally used as a proxy for foliar N content (Xiong et al., 2015). The SPAD measurement was taken from the first fully extended leaf of the main stem and was recorded as an average of three readings per plant. At the 4th week of assessment, 10 wheat plants per N and CO_2_ treatment were destructively sampled; in addition to plant height, tiller number, and chlorophyll content, dry weight and N content were measured.

### Bird Cherry-Oat Aphid Development and Fecundity

**Figure 1:**
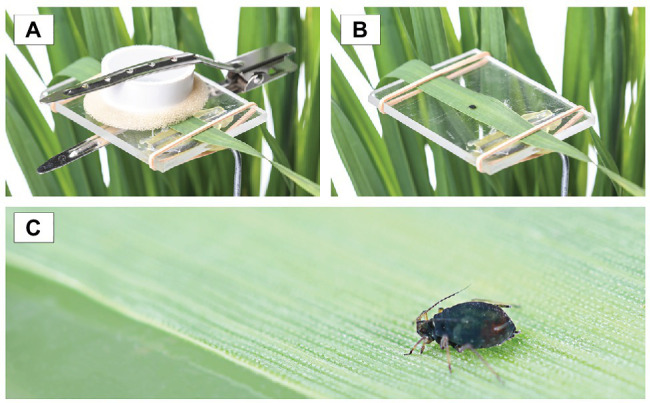
**(A)** The bird cherry-oat aphid was confined in a clip cage to study its development and fecundity, **(B)** transparent, acrylic platform used to support the leaf the bird cherry-oat aphid was reared on, and **(C)** close up of the adult bird cherry-oat aphid.

To evaluate aphid development and fecundity, a single adult bird cherry-oat aphid was placed on the second fully extended leaf of the main stem of each of the 14 replicates (insect-plant combination) for each treatment. Each aphid was confined in a clip cage (the top of the clip cage was covered with a fine mesh to allow transpiration) that was placed onto a transparent acrylic platform and secured to the plant by a hair clip (Figure 1; Trębicki et al., 2016). After 24 h, the female aphid and all its progeny, except for one nymph, were removed. Each individual nymph was assessed daily until adulthood. During each assessment, and for each of the 14 replicates, the instar number was recorded and the shed exuvia were removed. When the aphid reached adulthood, its fecundity was assessed by counting and removing the progeny every 24 h for at least 12 days. To evaluate the bird cherry-oat aphid performance, we calculated duration of the period from birth to the onset of both adulthood (development time) and reproduction (*d*), the mean nymph number per female over a period of time equivalent to the pre-reproductive period (*M*_d_), and the mean number of nymphs produced per aphid female over a 10-day period (*M*_10_). Additionally, we calculated the mean generation time (*T*_d_ = d/0.738), the intrinsic rate of natural increase [*r*_m_ = 0.738 (ln *M*_d_)/d], and the mean relative growth rate (RGR = *r*_m_/0.86) following calculations described by Wyatt and White (1977) and commonly used to assess aphid performance (Dáder et al., 2016; Trębicki et al., 2016; Moreno-Delafuente et al., 2020).

### Plant Carbon and Nitrogen Content

To determine aboveground nitrogen (N) and carbon (C) content, plants were oven dried (TD-150F, Thermoline Scientific, NSW, Australia) at 60°C for 72 h, and then finely ground (<0.5 mm) using a tissue lyser (Retsch MM300, Haan, Germany) prior to analysis by the Dumas combustion method at the University of Melbourne TrACEES Soil Node platform.

### Statistical Analysis

Two-way ANOVA was used to examine the effects of CO_2_, N and their interaction on wheat growth and quality and aphid development and performance variables. CO_2_ factor had two levels (aCO_2_ and eCO_2_) and N factor had three (low, medium, and high). In the case of a significant interaction of these factors (*p* < 0.05) on any of the measured variables, a simple main effects analysis was conducted. IBM SPSS for Mac was used to perform these analyses (IBM SPSS Statistics for Mac, Chicago, United States).

## Results

### Plant CO_2_ and Nitrogen Response

**Figure 2:**
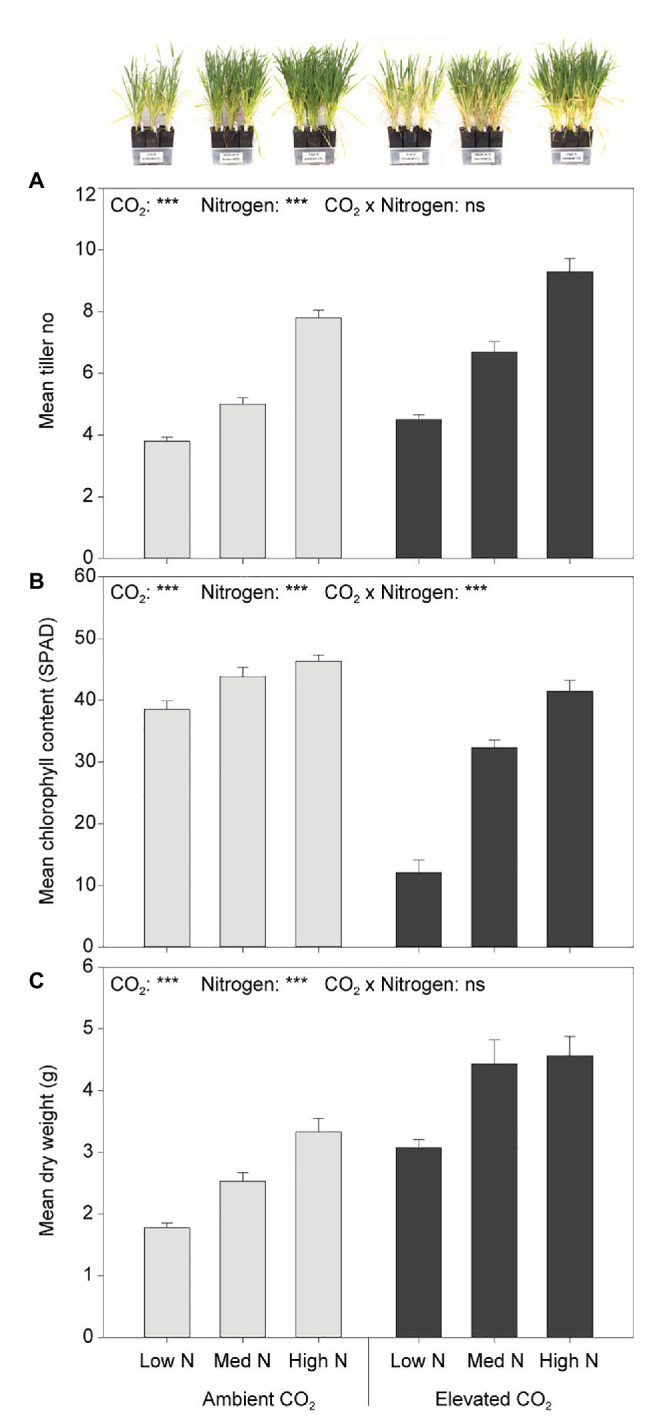
**(A)** Tiller number, **(B)** chlorophyll content, and **(C)** dry weight in response to CO_2_ and N treatments at the fourth week of plant assessment. ^***^p < 0.001. The error bars indicate the standard error of the mean (SEM). *N* = 10.

At the fourth week of plant assessment, eCO_2_ significantly increased tiller number by 23% (*F*_1,54_ = 34.308, *p* < 0.001) and biomass by 58% (*F*_1,54_ = 57.401, *p* < 0.001) when compared to aCO_2_. Greater nitrogen application rates also significantly increased tiller number (*F*_2,54_ = 133.263, *p* < 0.001; Figure 2A). Although the main effect of N was significant on dry weight (*F*_2,54_ = 21.214, *p* < 0.001), plant biomass did not significantly increase between the high and medium N levels (*p* = 0.170; Figure 2C). Furthermore, the leaf chlorophyll content was significantly decreased by 33% under eCO_2_ when compared to aCO_2_ (*F*_1,54_ = 129.603, *p* < 0.001), and significantly increased with greater nitrogen application rates (*F*_2,54_ = 76.915, *p* < 0.001). There was an interaction effect of CO_2_ and N on leaf chlorophyll content (*F*_2,54_ = 25.852, *p* < 0.001). The effect of N on leaf chlorophyll content was dependent on CO_2_ condition (Figure 2B). Under eCO_2_, greater nitrogen application rates significantly increased leaf chlorophyll content (*p* < 0.001). Nevertheless, under aCO_2_, the leaf chlorophyll content did not significantly differ between the high and the medium N levels (*p* = 0.747, 95% CI of the difference = −2.83 to 7.89) nor between the medium and low N levels (*p* = 0.053, 95% CI of the difference = −10.67 to 0.05), whereas it did between the high and low N levels (*p* < 0.01, 95% CI of the difference = 2.48–13.20; Figure 2B).

### Bird Cherry-Oat Aphid Development and Performance

The average development time of the bird cherry-oat aphid, measured as the duration of the period from birth to adulthood, ranged from 6.29 to 6.86 days across all treatments and was not significantly affected by N application nor CO_2_ condition. Elevated CO_2_ significantly increased the duration of the period from birth to the onset of reproduction (*d*; aCO_2_ = 7.78 and eCO_2_ = 8.317 days, *F*_1,76_ = 11.438, *p* < 0.001) and the mean generation time (*T*_d_; aCO_2_ = 10.52 and eCO_2_ = 11.27 days, *F*_1,70_ = 10.758, *p* < 0.01). Nevertheless, greater nitrogen application rates did not affect *d* (*F*_2,76_ = 1.109, *p* = 0.335) nor *T*_d_ (*F*_2,70_ = 1.277, *p* = 0.285).

**Figure 3:**
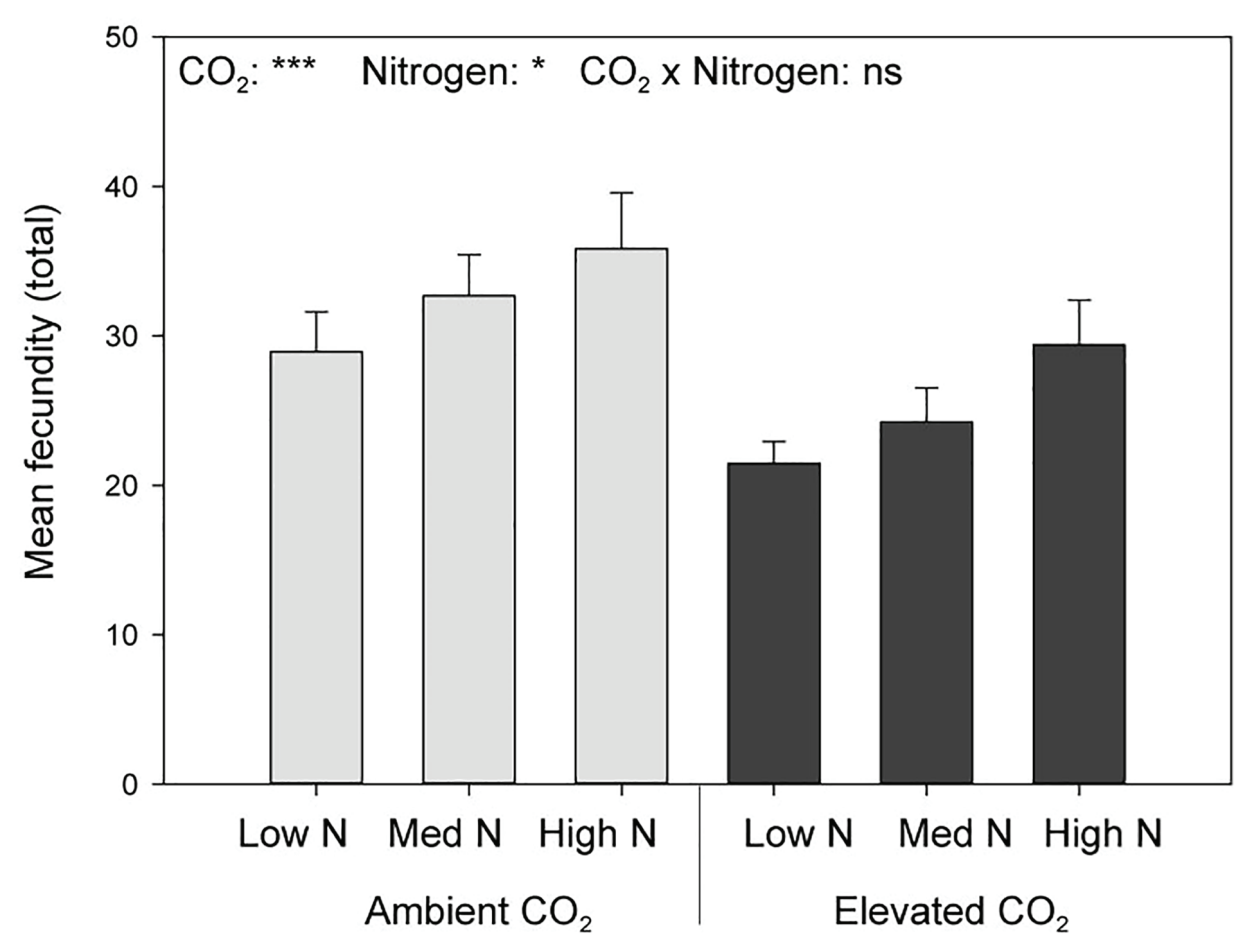
Total fecundity per female aphid in response to CO_2_ and N application. ^*^p < 0.05 and ^***^p < 0.001. The error bars indicate the SEM. *N* = 14.

**Figure 4:**
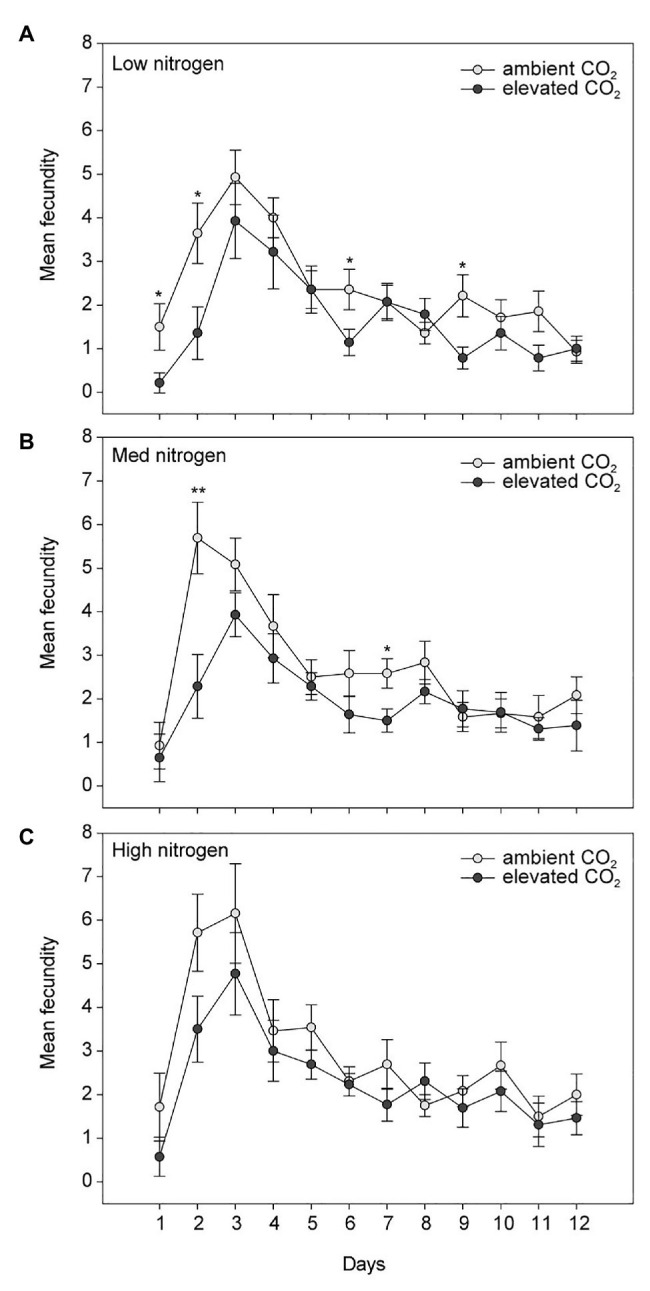
Daily fecundity in response to CO_2_ and **(A)** low, **(B)** medium, and **(C)** high N treatments. Day 1 indicates the day the aphid reaches adulthood. ^*^p < 0.05 and ^**^p < 0.01. The error bars indicate the SEM. *N* = 14.

Elevated CO_2_ significantly decreased the bird cherry-oat aphid total fecundity by 22%, calculated as the mean number of nymphs per aphid over a 12-day period starting from adulthood (aCO_2_ = 32.5 and eCO_2_ = 25 nymphs, *F*_1,70_ = 11.365, *p* < 0.001; Figure 3). It also decreased aphid daily fecundity when compared to aCO_2_, even if this difference was only significant on several assessment days for the low and medium N treatments (Figure 4). Greater nitrogen application rates significantly increased the aphid’s total fecundity (*F*_2,70_ = 3.806, *p* < 0.05; Figure 3), as well as the aphid daily fecundity (Figure 4). Moreover, eCO_2_ significantly decreased the mean nymph number per aphid over a period of time equivalent to the pre-reproductive period (*M*_d_) by 19% (*F*_1,70_ = 12.919, *p* < 0.001), and decreased the number of nymphs produced by each aphid from the onset of reproduction till the end of the assessment (*M*_10_) by 21% (*F*_1,70_ = 12.444, *p* < 0.001; Table 1). Greater nitrogen application rates significantly increased *M*_d_ (*F*_2,70_ = 3.530, *p* < 0.05). The intrinsic rate of natural increase (*r*_m_) and mean relative growth rate (RGR) were also significantly decreased by 13% under eCO_2_ when compared to aCO_2_ (*F*_1,70_ = 18.676, *p* < 0.001; Table 1).

**Table 1:** The bird cherry-oat aphid development and performance parameters in response to carbon dioxide (CO_2_) and nitrogen (N) treatments (mean ±SEM, *N* = 14).

Aphid parameter	CO_2_	Low N	Medium N	High N	CO_2_	N	CO_2_ × N
*d*	aCO_2_ eCO_2_	7.79 ± 0.21 8.57 ± 0.20	7.85 ± 0.19 8.29 ± 0.19	7.71 ± 0.16 8.08 ± 0.18	0.001^***^	0.335	0.500
*T*_d_	aCO_2_ eCO_2_	10.55 ± 0.29 11.67 ± 0.29	10.61 ± 0.28 11.15 ± 0.27	10.39 ± 0.25 10.95 ± 0.26	0.002^**^	0.285	0.483
*M*_d_	aCO_2_ eCO_2_	24.57 ± 1.89 19.77 ± 1.35	26.25 ± 1.54 20.38 ± 1.41	27.75 ± 2.30 23.33 ± 1.59	0.001^***^	0.144	0.910
*M*_10_	aCO_2_ eCO_2_	27.57 ± 2.43 21.15 ± 1.44	30.17 ± 2.23 22.85 ± 1.80	27.42 ± 2.30 33.17 ± 3.05	0.001^***^	0.035^*^	0.942
*r*_m_	aCO_2_ eCO_2_	0.31 ± 0.01 0.26 ± 0.01	0.31 ± 0.01 0.27 ± 0.01	0.32 ± 0.01 0.29 ± 0.01	0.000^***^	0.079	0.820
RGR	aCO_2_ eCO_2_	0.36 ± 0.01 0.30 ± 0.01	0.36 ± 0.01 0.32 ± 0.01	0.38 ± 0.01 0.34 ± 0.01	0.000^***^	0.079	0.820

d is the duration in days of the period from birth to the onset of reproduction, T_d_ is the mean generation time, M_d_ is the mean nymph number per female over a period of time equivalent to the pre-reproductive period, M_10_ is the mean number of nymphs produced per aphid female over a 10-day period, r_m_ is the intrinsic rate of natural increase, and RGR is the mean relative growth rate.

* *p* < 0.05;

** *p* < 0.01;

*** *p* < 0.001.

### Plant N and C Analysis

**Figure 5:**
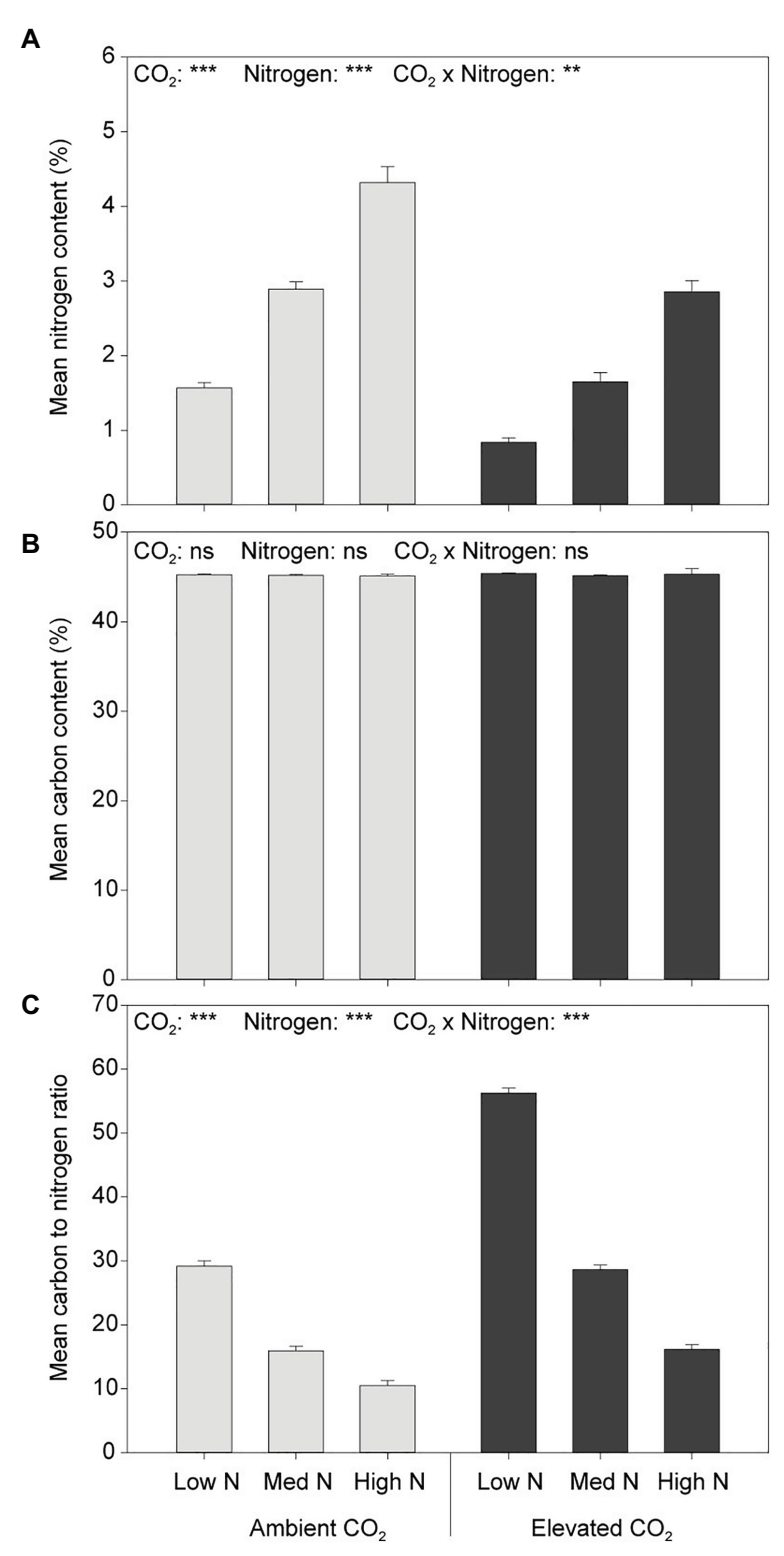
**(A)** N content, **(B)** C content, and **(C)** C:N in response to CO_2_ and N treatments. ^***^p < 0.001. The error bars indicate the SEM. *N* = 10.

Neither CO_2_ nor N had an effect on plant C content (Figure 5). Elevated CO_2_ significantly decreased the N content of aboveground biomass (both leaves and stems) by 39% (*F*_1,54_ = 168.848, *p* < 0.001) and increased the C:N ratio by 81% (*F*_1,54_ = 106.231, *p* < 0.001; Figure 5). Furthermore, greater N application rates significantly increased the N content (*F*_2,54_ = 245.163, *p* < 0.001) and decreased the C:N ratio of aboveground biomass (*F*_2,54_ = 139.953, *p* < 0.001).

## Discussion

Under future climate, the nutritional quality of wheat products will decrease. This can potentially be mitigated by increasing or optimizing fertilizer use, which in turn can increase aphid pest numbers thus the damage caused. To our knowledge, this is the first study which investigates the effects of different levels of CO_2_ and N fertilizer application on the development and performance of the bird cherry-oat aphid, which is a global pest and vector of viruses in wheat.

In our study, eCO_2_ significantly increased tiller number and aboveground dry biomass, which is consistent with previous findings (Bloom et al., 2010; Taub, 2010; Trębicki et al., 2016; Walker et al., 2017; Moreno-Delafuente et al., 2020). The observed eCO_2_-induced reduction in leaf chlorophyll content (a proxy for plant N content) has also been noted by others (Myers et al., 2014; Ryan et al., 2014; Dáder et al., 2016; Trębicki et al., 2016; Vassiliadis et al., 2018; Moreno-Delafuente et al., 2020). The mechanisms responsible for such a reduction are not fully understood, though Myers et al. (2014) proposed that it may be due to a combination of factors including carbohydrate dilution, slower N uptake in the roots and decreased transpiration-driven N flow, among others. It has also been suggested that nitrate assimilation is suppressed under eCO_2_ (Bloom et al., 2010).

Nitrogen fertilizer application often improves wheat yield (Sudderth et al., 2005; Belete et al., 2018; Xu et al., 2020). We observed that greater N application rates significantly increased tiller number, aboveground dry biomass, leaf chlorophyll content and plant N content of wheat, in agreement with different studies on wheat and other plant species (Chaturvedi, 2005; Sudderth et al., 2005; Walker et al., 2017). In our study, the positive effect of N fertilization on wheat N content was not sufficient to compensate for its reduction induced by eCO_2_. This suggests that the addition of N alone may not be able to sustain wheat N content under future CO_2_ conditions. Indeed, N application was not able to revert the eCO_2_-induced reduction in wheat grain protein concentration even under high N input (Walker et al., 2017).

Nitrogen is also an important macronutrient for aphid biological functions (Mattson, 1980). We found that the parameters used to evaluate aphid performance (*M*_d_, *M*_10_, *r*_m_, and RGR) were increased as the plant N content of the leaf tissue increased. Nevertheless, another study reported that the effect of eCO_2_ and high N inputs on *Solanum dulcamara* and *Amaranthus viridis* aphid populations was not dependent on the leaf C:N ratio (Sudderth et al., 2005). This supports that different insect-plant models respond differently to eCO_2_ (Ryalls and Harrington, 2016). Several studies have reported an increase in aphid fecundity (Jiang et al., 2018) and abundance (Ryan et al., 2015), while others a decrease in aphid fecundity under eCO_2_ (Newman et al., 1999; Awmack et al., 2004; Oehme et al., 2013; Ryan et al., 2014; Dáder et al., 2016; Trębicki et al., 2016; Moreno-Delafuente et al., 2020). Although we found that eCO_2_ significantly decreased the fecundity of the bird cherry-oat aphid, it was overall lower than that reported by Trębicki et al. (2016) on the same insect-plant model and under similar conditions (controlled plant growth chambers set at 20°C; aCO_2_ = 385ppm and eCO_2_ = 650ppm). Thus, the decrease in aphid fecundity under eCO_2_ could be attributed to the decrease in tissue N content, as well as to changes in the amino acid content in the phloem (Oehme et al., 2013; Ryalls and Harrington, 2016). We suspect that the lower fecundity under both aCO_2_ and eCO_2_ observed in our study when compared to that of Trębicki et al. (2016) may be caused by the rapid depletion of N by the wheat plants supplied with a single N application at sowing. Future studies would benefit from investigating the interactive effects of eCO_2_ and N application at different plant growth stages for different insect-plant models.

Furthermore, we found that eCO_2_ did not have a significant effect on aphid development time, measured as the duration of the period from birth to adulthood. This is consistent with a study using the same insect-plant model Trębicki et al. (2016), as well as others on different aphids and their predators (Awmack et al., 2004; Boullis et al., 2018; Jiang et al., 2018). However, a decrease in aphid development time was observed under eCO_2_ in *Brevicoryne brassicae* when reared on ornamental cabbages (Amiri-Jami et al., 2012). We found that greater N application rates did not significantly affect aphid development, duration of the period from birth to the onset of reproduction or mean generation time. Although the importance of N in aphid biological functions is widely reported (Hosseini et al., 2010; Zarghami et al., 2010), our study suggests that aphid development was not affected by N content or was potentially dulled by a stronger eCO_2_ effect.

We investigated the impact of CO_2_ levels, N application rates and their interactions on wheat growth and N content, and the development and performance of the bird cherry-oat aphid. Our study provides insights into aphid and wheat interactions under predicted future, higher CO_2_ climate, where management options to revert the CO_2_-induced reduction in grain protein content might be considered. It highlights the importance of considering the flow-on effects on insect pests when assessing strategies to address nutrient deficiency in cereals.

## Data Availability Statement

The raw data supporting the conclusions of this article will be made available by the authors, without undue reservation.

## Author Contributions

EC, SL, and PT planned and designed the research. EC and PT performed experiments and analyzed the data. EC wrote the manuscript, and SL and PT revised it. All authors contributed to the article and approved the submitted version.

### Conflict of Interest

The authors declare that the research was conducted in the absence of any commercial or financial relationships that could be construed as a potential conflict of interest.
